# Comparison of structure and immunogenicity of CVB1-VLP and inactivated CVB1 vaccine candidates

**DOI:** 10.21203/rs.3.rs-4545395/v1

**Published:** 2024-06-28

**Authors:** Saana Soppela, Zlatka Plavec, Stina Gröhn, Minne Jartti, Sami Oikarinen, Mira Laajala, Varpu Marjomaki, Sarah J Butcher, Minna M Hankaniemi

**Affiliations:** Tampere University; University of Helsinki; Tampere University; Tampere University; Tampere University; University of Jyvaskyla; University of Jyvaskyla; University of Helsinki; Tampere University

## Abstract

Coxsackievirus B1 (CVB1) is a common cause of acute and chronic myocarditis, dilated cardiomyopathy and aseptic meningitis. However, no CVB-vaccines are available for human use. In this study, we investigated the immunogenicity of virus-like particle (VLP) and inactivated whole-virus vaccines for CVB1 when administrated to mice via either subcutaneous or intranasal routes formulated with and without commercial and experimental adjuvants. Here, the potential of utilizing epigallocatechin-3-gallate (EGCG) as a mucosal adjuvant synergistically with its ability to inactivate the virus were investigated. EGCG had promising adjuvant properties for CVB1-VLP when administered via the parenteral route but limited efficacy via intranasal administration. However, intranasal administration of the formalin-inactivated virus induced high CVB1-specific humoral, cellular, and mucosal immune responses. Also, based on CVB1-specific IgG-antibody responses, we conclude that CVB1-VLP can be taken up by immune cells when administrated intranasally and further structural engineering for the VLP may increase the mucosal immunogenicity. The preparations contained mixtures of compact and expanded A particles with 85% expanded in the formalin-inactivated virus, but only 52% in the VLP observed by cryogenic electron microscopy. To correlate the structure to immunogenicity, we solved the structures of the CVB1-VLP and the formalin-inactivated CVB1 virus at resolutions ranging from 2.15 A to 4.1 A for the expanded and compact VLP and virus particles by image reconstruction. These structures can be used in designing mutations increasing the stability and immunogenicity of CVB1-VLP in the future. Overall, our results highlight the potential of using formalin inactivated CVB1 vaccine in mucosal immunization programs and provide important information for future development of VLP-based vaccines against all enteroviruses.

## Introduction

Coxsackieviruses B (CVBs) are classified within the *Enterovirus B* species and include six distinct serotypes (CVB1-CVB6) (https://ictv.global/report/chapter/picornaviridae/picornaviridae/enterovirus accessed 6.3.2024). Besides common cold symptoms, CVB infection can lead to severe outcomes especially in immunocompromised individuals. These complications may manifest for instance as meningitis^1^, pancreatitis^2^, and myocarditis^3^.

Mature CVB1 virions, like other enteroviruses, have 4 structural proteins termed viral protein 1–4 (VP1–4)^4,5^. They form an icosahedrally-symmetric capsid of about 30 nm in diameter with VP1 around the 5-fold, VP2 on the sides of the 2-fold and alternating VP2 and VP3 around 3-fold symmetry axes^4,5^. VP1–3 have similar tertiary p-sheet jelly-roll structures^4–6^. VP4 spans the inside of the capsid below the 5-fold and is embraced by VP1 N-terminal arms^4,5^. At the base of VP1, there is a hydrophobic pocket which normally contains a lipid factor^4,5^. For the virion maturation, a cleavage of a precursor protein VP0 to VP2 and VP4 needs to occur, a process for which RNA is thought to be essential^7^. Alternative CVB1 structural conformations are termed A-particles and empty particles. A-particles are formed upon an interaction of CVB1 with a host receptor^5^. This structural conformation allows for the exit of the genome to the host cell and is characterized by 4% expansion, formation of pores on the sides of the 2-fold axes, lack of a lipid factor, hydrophobic pocket collapse and loss of VP4^5^. Empty particles can adopt the mature virions or A-particles conformation but are lacking RNA^5,8^

Similar to empty particles, recombinantly produced Virus-Like Particles (VLPs) closely mimic the structure of infectious virions but lack genetic material. Production processes for VLPs exhibit rapid scalability compared to traditional culture methods^25,26^ and VLP vaccines can be generated for virus strains that do not readily grow in cell cultures. While VLPs may outperform traditional vaccine manufacturing, VLPs may lack equally robust immunogenicity of their virus-based counterparts and typically require the use of adjuvants for enhanced efficacy. Moreover, these vaccines may differ from native viruses in terms of antigenic properties, such as protein folding, post-translational modifications, and the presence of a viral genome.

Formalin inactivation of viruses is one of the key methods in the commercial production of human vaccines. When we applied optimized formalin inactivation protocol^9^ in production of a novel hexavalent vaccine targeting the six known CVB serotypes, we showed that the vaccine has an excellent safety profile in murine models and nonhuman primates and that it induces strong neutralizing antibody responses to the six serotypes in both species without an adjuvant^10^. However, treating the CVB1 virus too long with formalin, causes capsid disintegration and decrease of the virus concentration^9^. Also, we have shown that UV treatment destabilizes CVB1 virus^11^ and CVB1-VLP^12^ at elevated temperatures and lowers the immunogenicity of the vaccines, demonstrating the importance of treating the particles without destroying the conformation of the particle and the immunogenic epitopes. Although, our preclinical studies with CVB1-^12^ and CVB3-^13^ VLPs have shown that these VLPs induce high neutralizing and total IgG antibody levels, primarily of a Th2 type phenotype in the sera of immunized mice, additional ancillary components inducing mucosal immunity are also needed.

Here, we studied the potential of a known enterovirus capsid binding antiviral compound epigallocatechin-3-gallate (EGCG) as a vaccine adjuvant and its potential in eliciting immunity in systemic, mucosal and cellular compartments. EGCG is a type of catechin, a polyphenolic molecule abundant in green tea and it has been studied for its potential as both an antiviral agent and an adjuvant in vaccine formulations. Studies have shown that EGCG exhibits significant antiviral properties against hepatitis C^14,15^ and B^16^ viruses, influenza viruses^17–19^ and enterovirus-71^20^, by inhibiting viral replication. Recently it has also been shown to have antiviral properties against enteroviruses such as CVB1, CVB3 and CVA9^21^. Additionally, EGCG has been explored for its role as a vaccine adjuvant, demonstrating its ability to enhance the efficacy of an influenza subunit vaccine by inducing high levels of neutralizing antibodies, similar to alum adjuvant^22^. In addition to EGCG, we also tested a known adjuvant system 04 (AS04) that contains aluminium hydroxide and 3-O-desacyl-4’-monophosphoryl lipid A (MPLA) and is currently in clinical usage in VLP-based human papillomavirus vaccine and in other protein-based vaccines^23^.

The route of immunization determines the protective efficacy of vaccines and since mucosal sites are primary access points for CVBs, the induction of mucosal immunity by vaccination would prevent pathogen entry, infection and disease. According to our knowledge, no preclinical studies demonstrating the immunogenicity and safety of CVB1-VLP and inactivated CVB1 virus vaccines via the intranasal route have been published. Here, we demonstrated for the first time that CVB1-VLP can be taken up by immune cells when administrated intranasally and formalin-inactivated CVB1 is a promising mucosal vaccine for future clinical use. Based on the atomic resolution structures determined for the CVB1-VLP and formalin-inactivated CVB1 virus, we can further engineer beneficial mutations targeting the CVB1 capsid.

## Materials and Methods

### 1. Coxsackie B1 Virus-Like Particle (CVB1-VLP) and virus production and purification

A wild CVB1 field isolate (CVB1–10796, isolated from Argentina 1983^24^, kindly provided by Centers for Disease Control and Prevention, Division of Viral Diseases), gene bank accession number PP782006, was used in producing the formalin inactivated CVB1 vaccine and as a template for CVB1-VLP To produce CVB1-VLPs, a baculoviral transfer vector pOET5, which included distinct cassettes for the CVB1 VP0–3 polyprotein regulated by the polyhedrin promoter and the 3CD-protease controlled by the CMV promoter, was ordered from GenScript. The recombinant baculovirus was generated following the guidelines outlined in the FlashBAC baculovirus expression system (Oxford Expression Technologies) manual and utilizing the flashBAC ULTRA baculovirus genome. CVB1-VLPs were produced in High-Five insect cells with a multiplicity of infection (MOI) value of 1. Two separate purifications were performed for CVB1-VLPs using previously established purification method^12^. The VLPs were purified and stored without and with Tween80 (designated VLP_No-Tween80_ and VLP_Tween80_). Briefly, after harvesting the culture supernatant containing the VLPs, it was clarified by centrifugation (9610xg, 4°C, 30 min) and was filtered through a 0.2 μm filter. The VLPs were concentrated from the culture supernatant by Tangential Flow Filtration (TFF), utilizing 750 MWCO hollow fiber with an AKTA Flux system (Cytiva). The buffer was exchanged to 40 mM Tris pH 7.5, 10 mM MgCl_2_, 40 mM NaCl, with the same system. Impurities were removed from the preparation using HiTrap Q and SP columns (GE Healthcare), as well as CIMmultus QA and SO_3_ ion exchange chromatography (IEX) columns (BIASeparations). For VLP_No-Tween80_ additional baculovirus removal step was included utilizing POROS CaptureSelect BacuClear Affinity Matrix (Thermo Fischer Scientific). Endotoxins were removed with EndoTrap^®^ HD FPLC column (LIONEX), according to the protocol provided by the manufacturer. CVB1-VLP_No-Tween80_ and CVB1-VLP_Tween80_ were stored in Tris-buffered saline (40 mM Tris-HCl pH 7.4, 10 mM MgCl_2_, 0.2 M NaCl) without or with 0.1% Tween80 at −80°C.

CVB1 virus was produced in Green Monkey Kidney (GMK) cells that were kindly provided by the Finnish Institute for Health and Welfare (Virology Department). GMK cells were cultivated in MEM with Earle’s salts, 2 mM L-glutamine and sodium bicarbonate supplemented with 10% FBS and 1% penicillin-streptomycin (all from Sigma-Aldrich). The virus was propagated in GMK cells in multilayer (Falcon Cell culture Multi-Flask or Corning hyperflask M cell culture vessel) flasks using SFM4MegaVir protein-free medium (HyClone) without FBS for 2–5 days with MOI value of 5–10. Purification of the virus was performed similarly as previously^9^. Briefly, the viruses were recovered from the clarified GMK cell culture supernatant by 30% sucrose cushion pelleting (175,000 × g, 16 h at 4°C). The pellets were resuspended in phosphate-buffered saline (PBS)-0.1% Tween80 and were inactivated in 0.01% (v/v) formalin for 5 days at 37°C or with 524 μM EGCG for 28 days in RT. Virus inactivation was confirmed by the lack of infectious virus (after culturing the inactivated viruses in GMK cells) in TCID_50_ (median tissue culture infectious dose) end-point dilution assay (detection limit, 49 TCID_50_ units/ml). Briefly, first, a 6-fold CVB1 dilution series with eight replicates was made in 96-well plate. Viruses were diluted to Hanks balanced salt solution (HBSS) with 0.01M HEPES and 0.6% FBS (all from Sigma-Aldrich). 16 000 GMK cells in Minimum Essential Medium Eagle (MEM, Sigma-Aldrich) containing 5% FBS, were seeded on top of the virus dilutions. Viruses were allowed to infect cells for 46 hours in a humidified incubator at + 37°C with 5% CO_2_. 10μl of AlmarBlue Cell Viability Reagent (Invitrogen) was then added to the wells and the cells were incubated 2 hours in 37 degrees at CO_2_ incubator. Then, the fluorescence was measured with 560 nm excitation and 590 nm emission wavelengths using Envision UV/VUS (Perkin Elmer) multiplate reader. The fluorescent signal generated from the assay is proportional to the number of living cells in the sample. TCID_50_ calculation was based on Karber’s formula^25^.

### 2. CVB1-VLP and virus characterization

Purified VLPs and viruses were analyzed with mini-protean TGX stain-free precast gels (Bio-Rad). Virus and VLP proteins and impurities were assessed by densitometry analysis of SDS-PAGE gels using the ImageJ software^26^. VP1 and VP3 proteins were detected by Western blotting using a rabbit anti-CVB1–6 polyclonal antibody^9^. A mouse monoclonal anti-gp64 antibody (1:2000, Santa Cruz Biotechnology) was used to identify residual baculovirus in the purified CVB1-VLPs. IRDye 680RD goat anti-rabbit and IRDye 800CW goat anti-mouse secondary antibodies (1:20 000, LI-COR Biosciences) were used for visualization. Determination of VLP and inactivated virus total protein concentration, dynamic light scattering (DLS) analysis and transmission electron microscopy (TEM) imaging were performed as described^13^.

### 3.1. Cryo-Electron Microscopy Structural Determination of CVB1-VLP and formalin inactivated CVB1

Purified CVB1-VLP_Tween80_ stored at −80°C in Tris-buffered saline (40 mM Tris-HCl + 10 mM MgCl_2_ + 0.2 M NaCl + 0.1% Tween80, pH 7.3) at a concentration of 2 mg/ml and purified CVB1 virus treated by formalin (as described in section 1) and stored at −80°C were applied to glow-discharged Quantifoil R1.2/1.3 300 mesh copper grids with 2 nm carbon film. A 3 μl aliquot was incubated on the grid for 15 s and blotted for 1.5 s before vitrification in liquid ethane using a semi-automatic plunger Leica EM GP The chamber was set to 85% humidity, 22 °C.

CVB1-VLP_Tween80_ data was collected at SciLifeLab in Sweden using a Titan Krios equipped with Gatan K3 detector and operating at 300 kV. 40 frames per image were collected at 105 000 * nominal magnification resulting in 1.06 Å/pixel with a total dose of 63.368 e^−^/Å^2^. Images were taken between - 0.4 and −1.6 μm from focus at 0.2 μm defocus increments. Data collection details and statistics are described in Table 1 and Fig. 5. CVB1_formalin_ data was collected at CryoEM unit at the University of Helsinki using a FEI Talos Arctica equipped with a Falcon III detector and operating at 200 kV. 40 frames per image were collected at 150 000 * nominal magnification resulting in 0.97 Å/pixel with a total dose of 40 e^−^/Å^2^. Images were taken between - 0.1 and - 2.8 μm defocus. Data collection details and statistics are described in Table 1 and Fig. 5.

Images were processed in cryoSPARC v4.2.0, v4.4.1^27^ and Scipion v3.0.12^28^. Movies were motion and ctf corrected using Patch Motion Correction and Patch CTF Estimation from cryoSPARC^27^. A small set of particles was picked using Manual Picker in cryoSPARC and classified reference-free in 2D^27^. A representative class was used to generate a template for Template Picker in cryoSPARC^27^. A box size of 400 pixels was used to extract picked particles from the CVB1-VLP_Tween80_ dataset and 450 pixels box size for particles from CVB1_formalin_ dataset. Particles were iteratively classified in 2D after which classes containing particles were selected for further processing. Further processing differs for CVB1-VLP_Tween80_ and CVB1_formalin_ datasets. For the CVB1-VLP_Tween80_ dataset, a subset of randomly chosen 10 000 particles was used to generate 3 *ab initio* models^27^. The model with the highest number of different views created from 3 866 particles was used for homogeneous refinement in cryoSPARC using the full particle set^27^. Particles were exported to Scipion v3.0.12^28^ and classified in 2D and 3D using Relion 4^28–33^ Classification using Relion allowed for separation of compact and expanded particles. Particles were imported back to cryoSPARC in separate sets and locally CTF refined^31^. Non-uniform refinement was applied to improve the resolution. Parameters for non-uniform refinement were changed to correct for the curvature of the Ewald Sphere, optimize per-particle defocus and per-group CTF parameters^34^. For CVB1_formalin_ initial models used were final reconstructions of expanded and compact CVB1-VLPs_Tween80_ filtered to 30 A. The models were used as input to heterogeneous refinement in cryoSPARC resulting in the separation of particle populations corresponding to native CVB1 and CVB1 A-particles^66^. Particles were further CTF refined locally^70^ and underwent non-uniform refinement with correction for the curvature of the Ewald Sphere, optimized per-particle defocus and per-group CTF parameters^34^. Details of the image processing and refinement statistics for each data can be found in Table 1. The local resolution was estimated by utilizing the local resolution estimation tool in cryoSPARC^27^. The resolution of the final reconstructions was estimated according to the gold standard Fourier Shell Correlation of 0.143 threshold criterion and local resolution using Local Resolution job in cryoSPARC^27,35^.

### 3.2. Modeling and data deposition

The CVB1 atomic models PDB ID 7DPF and 7DQ4 were rigidly fitted into the compact and expanded CVB1-VLP_Tween80_ reconstructions, respectively, in UCSF ChimeraX v1.5^5,36,37^. The backbone was fitted using 3D refinement in Phenix v1.20.1 and the sidechains were manually adjusted in Coot 0.9.8.1^38,39^. Additional residues could be added based on the sequence of CVB1-VLP_Tween80_. Residues which had no corresponding density were deleted. The final models were validated using the MolProbity server and cryoEM validation in Phenix v1.20.1^39,40^. To obtain the difference map between modeled and unmodeled density, maps were created in UCSF ChimeraX v1.5 from the symmetrized models at 2.4 A resolution and subtracted using the subtract tool^36,37^. Table 1 shows the details of modeling and validation. The resolution of compact CVB1_formalin_ was insufficient for detailed modelling. Expanded CVB1-VLP_Tween80_ model was used as basis for modeling expanded CVB1_formalin_ structure using the same protocol. Raw data are deposited in the Electron Microscopy Public Image Archive (EMPIAR, https://www.ebi.ac.uk/empiar/) under the EMPIAR-xxxxx (CVB-VLP_Tween80_) and EMPIAR-xxxxx (CVB1 _formalin_) entry numbers, validated models to Protein Data Bank through One Dep system under PDB IDs 9FJC for compact CVB1-VLP_Tween80_, 9FJD for expanded CVB1-VLP_Tween80_ and 9FJE for expanded CVB1_formalin_. Density maps were deposited to the Electron Microscopy Data Bank under entry numbers 50497 for compact CVB1-VLP_Tween80_, 50 4 98 for expanded CVB1-VLP_Tween80_, 50 4 99 for compact CVB1_formalin_ and 50500 for expanded CVB1_formalin_. All the structure related figures were made in ChimeraX^41,42^ and CorelDraw2023.

### 4. Vaccine preparation, immunizations and sampling of mice

In the pilot study, CVB1-VLP_No-Tween80_ was used to evaluate the immune responses to VLP with different compounds. We wanted to analyze the immunogenicity of the VLP alone, VLP together with an adjuvant that is used in commercial protein-based vaccines (AS04) and VLP_No-Tween80_ with a potential new adjuvant EGCG. Vaccines were diluted to 2 μg per 150 μl dose using Tris buffer (20mM Tris-HCl pH 7.4 + 5 mM MgCl_2_ + 20mM NaCl) as a diluent. A mixture of AS04 components; 5 μg MPLA-SM VaccineGrade (Invivogen) dissolved in DMSO and 50 μg of 2% Alhydrogel (Invivogen) per dose was added prior to administration and left to bind in constant mixing for 10 min at room temperature. 6 μg of EGCG (purchased from Sigma-Aldrich and dissolved in distilled water in stock concentration 3 mg/mL) per dose was left to bind for 1 h at room temperature prior to immunization. 6-week-old female BALB/cJRj mice (Janvier Labs) were randomly divided into groups of 3 mice and were given a 150 μl dose subcutaneously (s.c.) on week 0 and 3. Mice were euthanized on week 6 and blood samples were collected to Microtainer SST Blood Collection Tubes (Becton Dickinson).

To study the mucosal immune responses for inactivated CVB1 virus and CVB1-VLP_Tween80_, vaccines were diluted to 2 μg in 25 μl per dose using PBS (pH 7.4) as a diluent. Inactivation of CVB1 was performed with formalin like described^9^ or with EGCG. For the CVB1-VLP_Tween80+EGCG_ group, 6 μg of EGCG was added to the preparation and incubated 2 h at room temperature prior to vaccinations. 9-week-old female BALB/cJRj were used. Six animals were randomly assigned per group and 2 μg of vaccine antigen was administered intranasally (i.n.) by pipetting a volume of 25 μl, to the nasal opening. To evaluate the mucosal immune responses, bronchoalveolar lavages were collected upon termination as described^41^, using PBS with Pierce Protease Inhibitor tablets (Thermo Fisher Scientific) and centrifuged 20 000 × g, + 4°C, 5 min before storing at −20°C until further use in enzyme-linked immunosorbent assays (ELISA).

All mice were housed in a pathogen-free environment in individually ventilated cages and food and water was provided *ad libitum*. Laboratory animal usage permission (Regional State Administrative Agency, Pirkanmaa, Finland; decision number ESAVI/1408/2021) covers all animal experiments conducted in this study.

### 5. Antigen specific IgG, IgG subtype and IgA antibodies post vaccination

Antigen-specific IgG, IgG1, IgG2a and IgA antibodies were determined with indirect ELISA as previously described^13^. HRP conjugated anti-mouse secondary antibodies IgG (1:3000), IgG1 (1:1000) and IgG2a (1:1000) from Invitrogen were used to detect antigen specific IgG and IgG subtype antibodies in the serum. In addition, HRP-conjugated goat anti-mouse IgA (1:1000, Invitrogen) was used to detect IgA antibodies in bronchoalveolar lavage (BAL). Positivity cut-off value for the end-point titer was determined with buffer control mice OD values with average OD + 3*SD.

The final antibody dilution above the experimental cutoff value was determined as the end-point titer for the antibody. End-point titer values for each sample were plotted in graphs expressing the geometric mean titer (GMT) of each experimental group. GMT calculation was performed by taking the antilog of the mean of the log titer transformation. The positivity cutoff value was given an arbitrary value of half of the titer of which the 2-fold serum or BAL dilutions were started (positivity cutoff being 1:200 dilution for serum and 1:5 for BAL).

### 6. Neutralizing antibodies

To analyze the CVB1 neutralizing capacity of the vaccinated mice sera and BAL samples, neutralizing antibodies against a live virus were measured by cell viability based micro-neutralization assay using GMK cells. First, two-fold serial dilution series of serum and BAL were prepared in duplicate in HBSS with 0.01 M HEPES and 0.6% FBS (all from Sigma-Aldrich) into a 96-well plate. 50 × TCID_50_ units of the CVB1 virus was added per well and incubated at 37°C for 1 h to allow neutralizing antibodies to bind the viruses. Then, 16 000 GMK cells in Minimum Essential Medium Eagle (Sigma-Aldrich) containing 5% FBS, were seeded on top of the pre-incubated virus and serum dilutions. After 46 h of incubation in a humidified incubator at + 37°C with 5% CO_2_, 10μl of AlamarBlue Cell Viability Reagent (Invitrogen) was added to the wells and the fluorescent signal generated from the assay was measured like described for TCID_50_ assay. Neutralization curves were plotted in GraphPad Prism 9.0.0 Software using 4-PL curve fitting constrained to 0% at the bottom and to 100% at the top and the inhibitory concentration value was determined from each curve. The fifty percent neutralizing titer designated as inhibitory concentration (IC_50_) in Prism 9.0.0 was defined by the midpoint of the sample-specific neutralization curve. The geometric means of the IC_50_-titers (with geometric standard deviation) were plotted on the graphs presenting the neutralizing antibody responses.

### 7. T-cell specific cytokine secretion from stimulated splenocytes

Mouse FluoroSpot^PLUS^ assay kit (Mabtech) was used to measure the levels of IFN-γ, IL-2, and TNF-α secreted from lymphocytes extracted from spleens of the vaccinated mice. Upon termination, the spleens were collected and splenocytes were extracted as follows: organs were minced through sterile 40 μm cell strainers (Thermo Fisher Scientific) and washed with PBS + 1%FBS + 2 mM EDTA. Cells were centrifuged and red blood cells were lysed with 1 ml of ACK buffer (Gibco) for 1 min. Lysed cells were removed by washing with PBS + 1% FBS + 2 mM EDTA. Cells were seeded in duplicates with the concentration of 2.5 × 10^5^/well in RPMI1640 GlutaMAX Medium (Thermo Fisher Scientific), supplemented with 25 mM HEPES and penicillin-Streptomycin (1:100) and 10% FBS (all from Sigma-Aldrich). Splenocytes were stimulated *in vitro* with CVB1 (1 μg/well, for 18 h in an incubator at 37°C, 5% CO_2_). Concanavalin A (Sigma-Aldrich) (2 μg/well) and culture medium were used as positive and negative controls. The following experimental procedures were carried out in accordance with the manufacturer’s user manual. The spot-forming cells were counted with Mabtech IRIS ELISpot/FluoroSpot reader (service by MabTech, Stockholm, Sweden). The positivity cut-off value for each mouse was determined individually as the average count of spots in the negative control wells + 3*SD. The resulting frequencies of responsive cells were reported as the number of spot-forming units per 10^6^ splenocytes.

### 8. Statistical analyses

Statistical analyses were done with GraphPad Prism 9.0.0. Immunological data was treated as non-parametric and the comparison between multiple groups was done using Kruskal Wallis t-test followed with Dunn’s Multiple Comparison test.

## Results

### Characterization of VLP_No-iween80_ and VLP_jWeen80_ and binding capacity of EGCG to the VLPs

**Figure 1:**
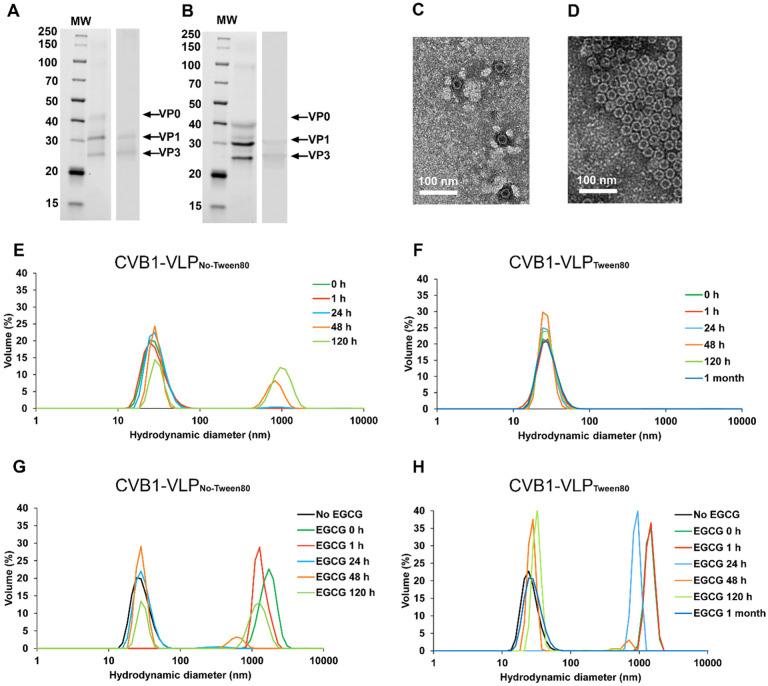
CVB1-VLP_No-Tween80_ and CVB1-VLP_Tween80_ characterization, stability and binding capacity to EGCG. SDS-PAGE and Western Blotting of purified A) CVB1-VLP_No-Tween80_ and B) CVB1-VLP_Tween80_. The left panel in A and B shows the stainfree total protein staining of the purified CVB1-VLP_No-Tween80_ and CVB1-VLP_Tween80_. In the right panels of A and B VP1 and VP3 capsid proteins were detected by Western blot stained with a polyclonal anti-CVB1–6 rabbit antibody. VLP capsid proteins are indicated with arrows. B) Transmission electron micrographs of the negatively-stained purified C) VLP_No-Tween80_ and D) CVB1-VLPTween80 with 25 000x magnification. Dynamic Light Scattering (DLS) analysis of E) CVB1-VLP_No-Tween80_ and F) CVB1-VLP_Tween80_ stored at room temperature. DLS analysis of G) CVB1-VLP_No-Tween80_ and H) CVB1-VLP_Tween80_ incubated with EGCG at room temperature.

Our previous studies have demonstrated that the use of Tween80 during purification significantly enhances the stability and yield of CVB1 and CVB1-VLP preparations^9,11,12^. Given EGCG’s antiviral properties in the absence of Tween80^20^, separate VLP preparations were purified using previously established purification method^12^ and were stored without and with Tween80 (designated VLP_No-Tween80_ and VLP_Tween80_). The VLP purification in the presence of Tween80 led to 5.2 times better pure protein yield compared to preparation that did not include Tween80 (11.4 mg/L pure VLP_Tween80_ and 2.2 mg/L pure VLP_No-Tween80_). SDS-PAGE analysis and densitometric analysis confirmed the purity of the VLPs, resulting in ≥ 95% pure preparations for VLP_No-Tween80_ (Fig. 1A) and for VLP_Tween80_ (Fig. 1B). Electron micrographs of negatively-stained preparations showed intact particles with an average particle size of 30 nm (Fig. 1C and D for VLP_No-Tween80_ and for VLP_Tween80_ respectively). Subsequently, we determined the stability of the VLPs in room temperature as well as the binding capacity of the antiviral compound, EGCG, to the VLPs in the absence and in the presence of Tween80 compound. DLS analysis of the freshly prepared VLP_No-Tween80_ showed a homogeneous monodisperse distribution, 100% of the particles had an average hydrodynamic diameter of 29.1 nm (± 0.44) (Fig. 1E). However, stability testing over 120 h in Tris-buffer at RT revealed particle aggregation, with 30.9% (± 2.4) of VLP_No-Tween80_ particles aggregating after 48 h, representing an average particle population of 829 (± 29.25) nm in diameter and 54.6% (± 0.1) of VLP_No-Tween80_ particles had aggregated after 120 h, representing an average particle population of 1021 (± 11.58) nm in diameter (Fig. 1E). Also, VLP_Tween80_ was analyzed for stability with DLS. 100% (± 0.3) of the freshly measured VLP_Tween80_ had an average hydrodynamic diameter of 28.13 nm (± 0.62) and since this result had not changed at 120 h’ time point, we followed the stability of VLP_Tween80_ until one month and by that time, the proportional amount or the average hydrodynamic diameter had not changed significantly, 100% (± 0.3) of the particles representing 29.5 nm (± 0.62) (Fig. 1F). This demonstrates that Tween80 significantly improves the solubility and stability of CVB1-VLP.

DLS was exploited in determining EGCG’s binding capacity to the VLPs. DLS analysis demonstrated that 100% of VLPs_No-Tween80_ agglomerated in the presence of EGCG up to one hour, resulting in an average hydrodynamic diameter of 1714 nm (± 154.4) immediately (at 0 h, Fig. 1G). However, after 24 h incubation at RT, 96.7% (± 0.26) of the VLP particles had detached from the agglomerate with an average particle population of 30.6 (± 0.82) nm in diameter (Fig. 1G). After 48 h, the proportion of VLP_No-Tween80_ particle population was similar in VLP samples incubated with or without EGCG, indicating time-dependent and reversible binding of EGCG to CVB1-VLP particles that do not contain Tween80 (Fig. 1E and G). Furthermore, DLS analysis revealed that EGCG caused measurable agglomeration of VLP_Tween80_ from 0–24 h, with particle detachment from the agglomerate occurring after 48 h, when 91.8% (± 3.4) of VLP_Tween80_ particles had an average hydrodynamic diameter of 27.1 nm (± 1.45) (Fig. 1H).

### Adjuvanticity of EGCG on CVB1-VLP via parenteral immunization route

**Figure 2:**
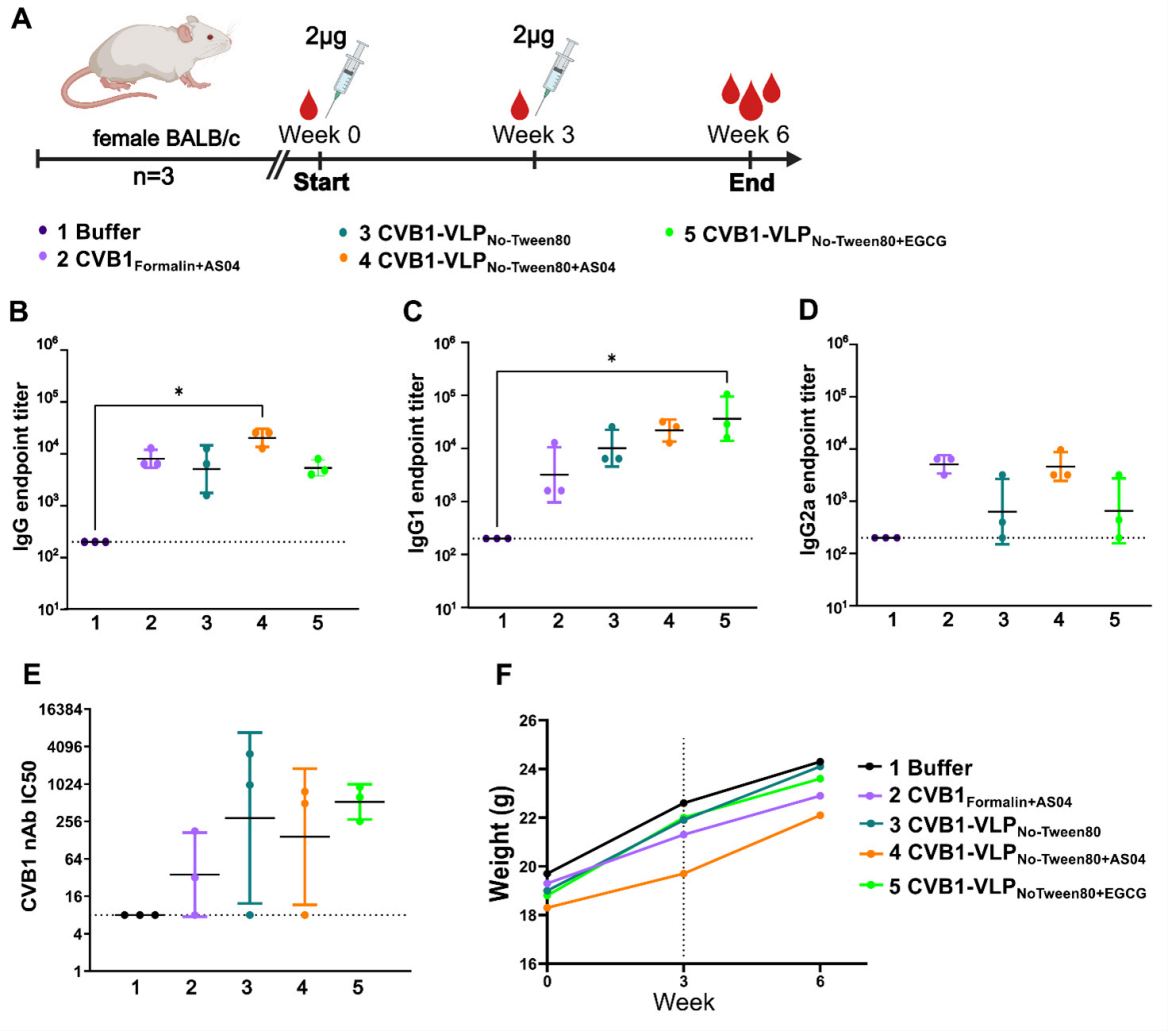
Pilot animal study. A) Immunization schedule. B) Vaccine antigen-specific IgG, C) IgG1 and D) IgG2a antibody end point titers at week 6 after two subcutaneous immunizations with 2 μg vaccine antigen determined with indirect ELISA. Titers below 200 were assigned as negative result. E) CVB1 neutralizing antibodies determined with microneutralization assay. Titers below 8 were assigned as a negative result. A-E). Data is shown as geometric mean with geometric SD. F) Average weights of animals in each experimental group. Dashed line indicates the time point for booster vaccination.

First, we assessed the immunogenicity of the CVB1-VLP_No-Tween80_ administrated *via* the subcutaneous immunization route since this administration route mimics the routine vaccination approach in humans. The humoral immune response elicited by CVB1-VLP_No-Tween80_ in combination with the well-established parenteral adjuvant system 04 (AS04) or with the potential adjuvant EGCG was analyzed, as illustrated in Fig. 2. CVB1-VLP_Tween80_ was excluded from this study because we wanted to analyze the immunogenicity of CVB1-VLP in the presence of EGCG without other compounds. Formalin-inactivated virus vaccine was included in the study as reference vaccine, because we have previously demonstrated that it induces robust immune responses when administrated parenterally^9–11,42,43^. We chose to use neutralizing antibodies measured against live CVB1 as the primary comparison point for efficiency of vaccine candidates and we evaluated the total antigen-specific IgG antibodies, alongside comparing the IgG1 subtype as an indicator of humoral immune response and IgG2a subtype as an indicator of cellular immune response.

The highest levels of antigen-specific IgG antibodies were observed in the CVB1-VLP_No-Tween80+AS04_ group (Fig. 2B). Interestingly, IgG1 antibody titers were most noticeable in mice sera immunized with CVB1-VLP_No-Tween80+EGCG_, suggesting a potential enhancement of antigen-specific humoral immune responses by EGCG (Fig. 2C). Although there were no statistically significant differences observed in neutralizing antibodies, the CVB1-VLP_No-Tween80+EGCG_ exhibited the least variance, as seen in Fig. 2E. Furthermore, mice in all the vaccine groups were healthy as weight gain continued as expected (Fig. 2F), and there were no signs of inflammation at the injection site. Due to ethical considerations, the number of animals used in the pilot study was low, and the results had no clear statistical significance. However, the study demonstrated that EGCG neither compromised nor hindered the antigen-specific immune response. The results from the pilot animal study supported the usage of EGCG as vaccine adjuvant and based on these results, we decided to further study the applicability of EGCG in CVB1 virus inactivation and as mucosal adjuvant.

### The potential of EGCG in producing inactivated CVB1 virus vaccine

**Figure 3:**
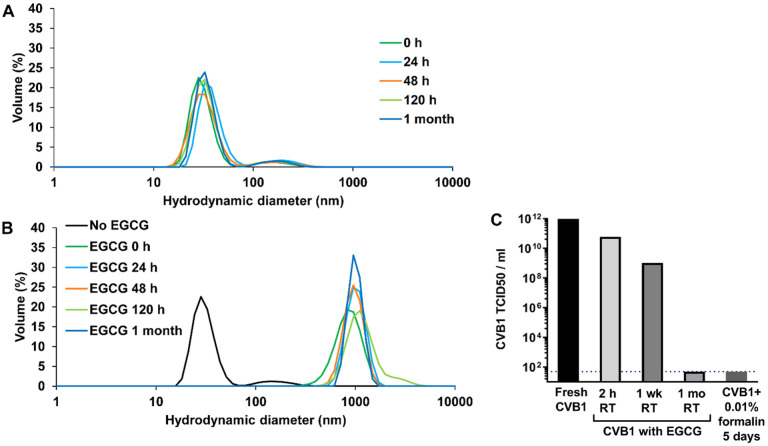
Characterization of CVB1 virus. A) DLS analysis of purified CVB1 virus stored at room temperature (RT) for 1 month. B) DLS analysis of purified CVB1 virus incubated with EGCG at RT for 1 month. C) To confirm that the virus was inactivated with EGCG and unable to replicate, TCID_50_-assay was carried out.

Although formalin inactivated viruses are in wide use in clinical vaccines, formalin inactivation has been shown to cause destruction of some immunogenic epitopes compared to live vaccines^9,44^. Therefore, we tested inactivation of CVB1 using EGCG incubation as a potential replacement for formalin (Fig. 3). Dynamic light scattering (DLS) analysis of active CVB1 virus revealed that the virus can be stored at room temperature (RT) up to 1 month without significant change in the average particle diameter or volumetric distribution. At day 0, 92% (± 1.2) of virus-particles had an average hydrodynamic diameter of 30.7 nm (± 3.28) whereas after 1 month storage in RT, 91.1% (± 2.0) of the particles had an average hydrodynamic diameter of 33.4 nm (± 1.6) (Fig. 3A).

DLS analysis of CVB1 incubated with EGCG at RT showed immediate aggregation of the virus, and this seemed to be irreversible effect as the measurement result did not change during one month following period (Fig. 3B). However, based on infectivity measurement with TCID_50_ assay, CVB1 was found to be completely inactivated by EGCG only after one month incubation at RT, achieving similar levels of infectivity reduction as formalin inactivation after 5 days (Fig. 3C). Considering that enteroviruses typically infect through the respiratory tract or gastrointestinal tract mucosa, establishing sterilizing immunity at the site of viral entry would be advantageous. Therefore, our next objective was to evaluate the immunogenicity of EGCG inactivated CVB1 virus and CVB1-VLP adjuvanted with EGCG via the intranasal immunization route.

### Adjuvanticity of EGCG via intranasal immunization route

The effect of EGCG as a mucosal adjuvant was assessed by administering vaccines intranasally as nasal drops. We aimed to compare formalin and EGCG inactivated CVB1 vaccines (CVB1_egcg_ and CVB1_Formalin_) to CVB1-VLP_Tween80_ alone or formulated with EGCG (CVB1-VLP_Tween80_ and CVB1-VLP_Tween80_+_EGCG_). To evaluate mucosal immune responses, bronchoalveolar lavages (BAL) were collected from the mice at the termination point of the study. Particularly, antigen-specific secretory IgA and IgG exhibited significant increases in the CVB1_Formalin_ group but conversely these responses were low in the CVB1_EGCG_ group (Fig. 5B–D), where the virus had aggregated when inactivated with EGCG. A similar trend can be seen in the CVB1 neutralizing antibodies from BAL samples, although no statistical significance was obtained. CVB1-VLP groups elicited low or negligible mucosal responses, as illustrated in Fig. 5B–D. Cellular immunogenicity (IFN-γ, IL-2, and TNFa secretion levels) induced by the vaccines was measured from splenocytes extracted from the vaccinated animals and stimulated with active CVB1. Interestingly, CVB1_Formalin_ and CVB1_egcg_ groups demonstrated heightened cytokine secretion patterns compared to CVB1-VLP groups that displayed limited to no cellular responses, as outlined in Fig. 5E–G. While EGCG appears to increase cytokine secretion in some animals, there is greater variability among animals in the EGCG-inactivated group compared to the formalin-inactivated virus group.

Neutralizing antibodies, antigen-specific IgG, and IgG subtypes IgG1 and IgG2a were quantified from the termination serum. CVB1_Formalin_ exhibited significantly higher responses across all antibody types, indicative of a relatively balanced immune response, as depicted in Fig. 5H–K. On the contrary, CVB1_egcg_ elicited a weak neutralizing antibody response and a diminished IgG1 response, suggesting inhibition of humoral immune response. Despite this, the IgG2a antibody response showed modest improvement, aligning with the observed cytokine secretion patterns of CVB1_EGCG_ group. Compared to the CVB1_Formalin_, the CVB1-VLP_Tween80_ group elicits a mediocre humoral immune response, indicating that VLPs can be taken in for antigen processing from the mucosa but not as efficiently as formalin-inactivated vaccine. However, formulating VLP with EGCG (CVB1-VLP_Tween80+EGCG_) seems to inhibit the intake of VLPs further. These results suggest that EGCG does not enhance mucosal antigen intake; rather, it exhibits inhibitory effects as a mucosal adjuvant. While there is a slight elevation in Th1 response in certain animals, indicating cellular response induction, it is not consistently reproducible. However, formalin inactivated CVB1 without added adjuvants raise a balanced immune response both in mucosal site and systemically, when administrated intranasally.

### CryoEM analysis of CVB1-VLPs_rween80_ and CVB1foimalin

**Figure 4:**
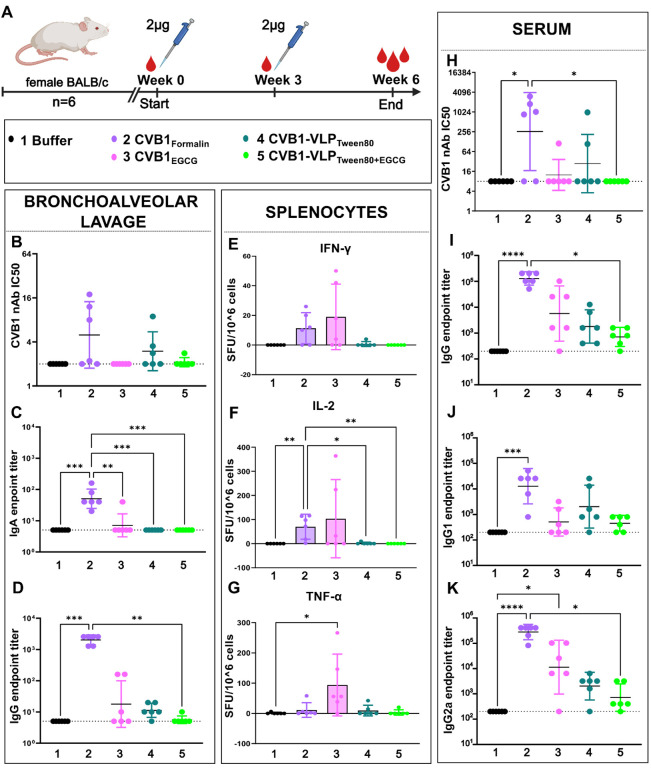
Intranasal immunization. A) Immunization schedule. B) CVB1 neutralizing antibodies, C) Vaccine antigen-specific IgA and D) IgG antibody end point titers in bronchoalveolar lavage at week 6 after two intranasal immunizations. E) IFN-g F) IL-2 and G) TNFα secreting splenocytes after stimulation with CVB1. H) CVB1 neutralizing antibodies, I) antigen-specific IgG, J) IgG1 and K) IgG2a antibodies from termination serum. Antigen specific antibodies were determined with indirect ELISA. Titers below 200 were assigned as negative. Neutralizing antibodies determined with microneutralization assay. Titers below 8 were assigned as negative. Shown as scatter dot plots with geometric mean and geometric SD. Cytokine secreting splenocytes were determined with FluoroSpot assay and shown as spot-forming units (SFU)/10^6^ splenocytes, mean with SD.

**Figure 5:**
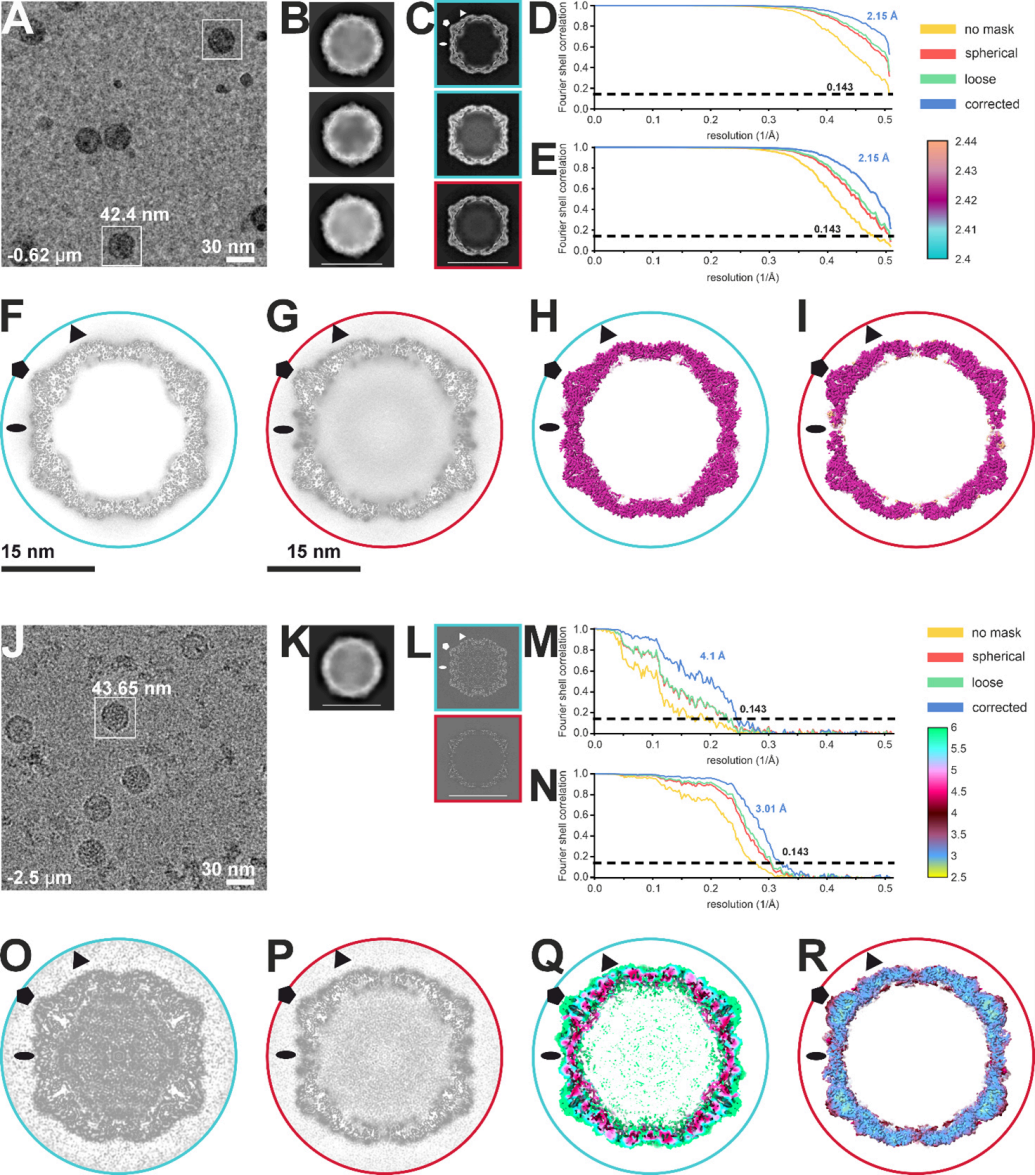
CryoEM data collection and image processing. Panels A-I are related to CVB1-VLP_Tween80_ dataset and J-R to CVB1_formalin_ dataset. A) and J) Representative micrograph from each dataset. Scale bar is shown on the figure. An example of picked particles is boxed in white with a corresponding box size. B) and K) Representative 2D classes after final 2D classification of particles chosen for further processing obtained by Relion 4 in Scipion (B) or cryoSPARC (K). Scale bar of 30 nm is shown. **C)** and L) 3D classes obtained by Relion 4 in Scipion (C) or heterogeneous refinement in cryoSPARC (L). Particles in classes boxed in blue were chosen for further processing together within each dataset as they correspond to compact particles. Particles contributing to class boxed in red correspond to expanded particles. Symmetry axes are marked with elipse (2-fold), triangle (3-fold) and pentagon (5-fold). Scale bar of 30 nm is shown on the bottom figure. D,E,M,N) Fourier shell correlation (FSC) curves for compact (D) and expanded (E) CVB1-VLP_Tween80_ and compact (M) and expanded (N) CVB1_formalin_ final reconstructions obtained by non-uniform refinement in cryoSPARC v4.2.0 (VLP) and v4.4.1 (formalin inactivated)^66^. The global resolution of CVB1-VLP_Tween80_ structures is estimated at 2.15 Å as the estimation reaches the Nyquist limit. The legend for FSC curves calculated with different masks applied is on the right. F,G,O,P) Central plane views of the final reconstructions for compact (F,O) in blue and expanded (G,P) in red. Symmetry axes are marked with elipse (2-fold), triangle (3-fold) and pentagon (5-fold). Scale bars of 15 nm are shown below the figures. H,I,Q,R) Local resolution of final reconstructions for compact (H,Q) in blue and expanded (I,R) in red shown as a central slice of surface representation at 2.5*σ* above mean (H,I,Q) or 5.5 *σ* above mean (R). Local resolution values legend is shown in Ångstrom. Symmetry axes are marked with elipse (2-fold), triangle (3-fold) and pentagon (5-fold).

**Figure 6:**
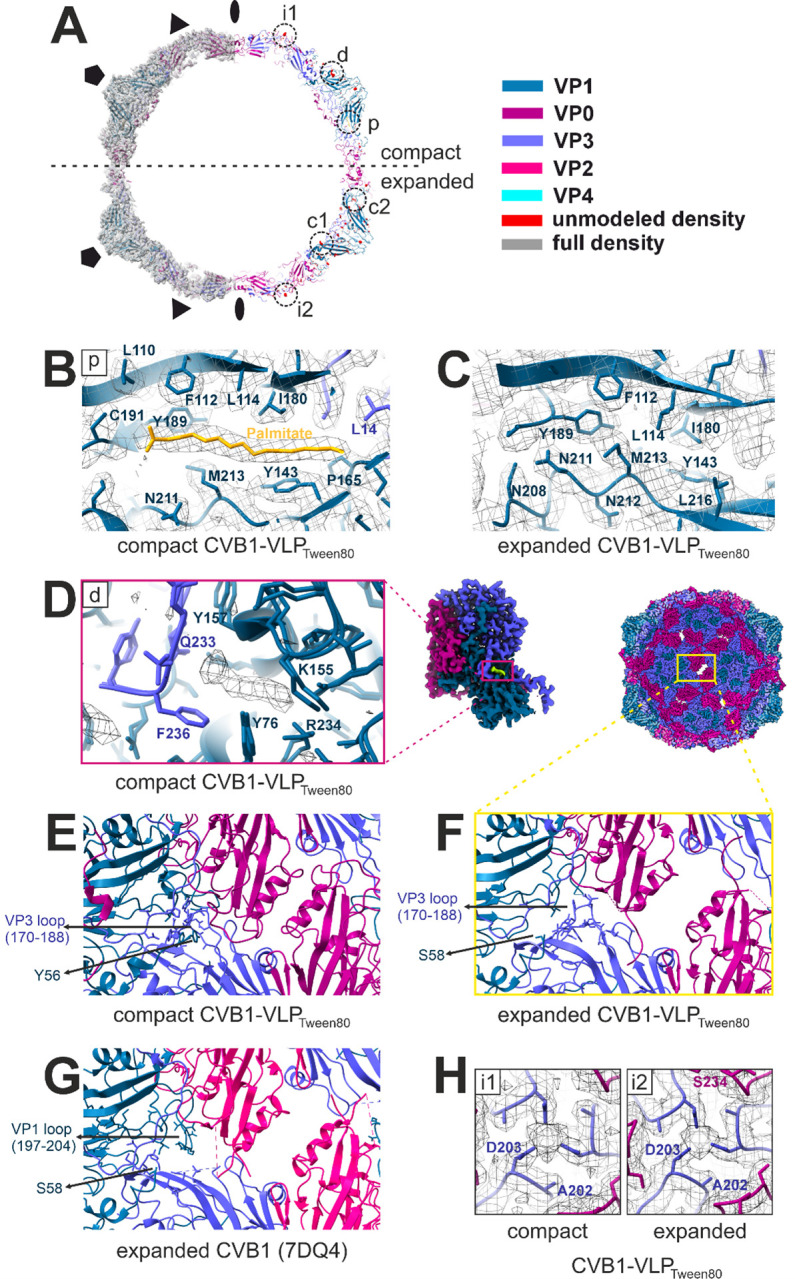
Final reconstruction analysis of compact and expanded CVB1-VLP_Tween80_ obtained by cryoEM and single-particle reconstruction. A) Final reconstructions shown as surface at 2.5σ above mean with fitted model (left) and the atomic model with unmodeled density in red (right). Unmodeled densities and positions of interest are marked with circles and are coded with small letters. B) Focused view on the hydrophobic pocket (at position p on A) housing a lipid factor represented as palmitate in compact CVB1-VLP_Tween80_. Density map is shown as mesh at 2.5σ above mean. C) Focused view on the expanded CVB1-VLP_Tween80_ density map on the site which corresponds to position in B). The pocket in the expanded particles is collapsed and there is no density corresponding to a lipid factor. Density map is shown as mesh at 2.5σ above mean. D) Focused view on the interprotomer pocket (position d on A) of compact CVB1-VLP_Tween80_ and native CVB1 (PDB ID 7DPF) located at the interface of two asymmetric subunits between VP1 and VP3 as shown on the right. Unmodeled density present in the interprotomer pocket of CVB1-VLP_Tween80_ is shown as mesh at 2.5σ above mean on the left. On the right one asymmetric subunit is shown as surface at 2.5σ above mean with the unmodeled density in green. E) - G) Focused views on the helices around the 2-fold symmetry axis and the VP3 loop of compact (E) and expanded (F) CVB1-VLP_Tween80_ and expanded CVB1 (G) at position as shown in F). H) Focused view down the 3-fold symmetry axis. The density map is shown as mesh at 2.5σ above mean showing the unmodeled density between Ds of VP3 (positions i1 and i2 in A).

To further understand the immunogenic differences between the preparations, we examined the structures of the CVB1-VLP_Tween80_ particles and CVB1_formalin_ preparations used in intranasal vaccinations (Fig. 4). 13 860 micrographs of CVB1-VLP_Tween80_ were collected and 226 223 particles were picked (Table 1, Fig. 5A,B). Through classification steps, particles were later separated into 2 distinct classes, compact CVB1-VLP_Tween80_ structurally corresponding to native CVB1 virions with VP0, 1 and 3 (PDB ID 7DPF) and expanded CVB1-VLP_Tween80_ similar to CVB1 A-particles (PDB ID 7DQ4, Table 1, Fig. 5C,F–I and 6A–C,E,F)^5^. Particle distribution is 48–52% in favor of the expanded particles (Table 1). Particle sets were processed in cryoEM and Scipion to 2.15 Å resolution reaching Nyqvist (Table 1, Fig. 5D,E,H,I). The high resolution and quality of the reconstructed data can be observed in Fig. 6H and S1A. At the current resolution, an ion coordinated between three D203 at the 3-fold (Fig. 6H) can be observed in both expanded and compact forms. In the expanded CVB1-VLPs_Tween80_, a forked density is present above cysteins which could be additional ion densities and in the compact CVB1-VLP_Tween80_ two conformations of VP3 R232 are resolved (Figure S1A). Local resolution estimation gives the lowest resolution of 2.44 A at the flexible regions on the sides of the 2-fold outside and inside the capsid (Fig. 5H,I).

Compact CVB1-VLP_Tween80_ density map fits well the model of native mature CVB1 taken from PDB ID: 7DPF (Fig. 6A–E,H)^5^. The structure and position of VP0 in VLPs corresponds to VP2 and VP4 in mature virions, however lacking a 39 residues long section on the inside of the capsid and missing an N-terminus and myristoyl group (Table 1, Fig. 6A). Furthermore, N-terminus arms embracing the VP4 in the native CVB1 could not be modelled prior to residue 56 in the CVB1-VLP_Tween80_ (Table 1, Fig. 6E). Other characteristic structural features of native CVB1 are seen, such as the VP1 around the 5-fold (Fig. 6A), lipid factor inside the hydrophobic pocket (Fig. 6B), VP0 helices on the sides of the 2-fold symmetry axis (Fig. 6E) and VP0 and VP3 alternating around a 3-fold (Fig. 6H). There is a molecule occupying the interprotomer pocket in CVB1-VLP_Tween80_ not present in PDB ID 7DPF, as shown in Fig. 6A,D. Residues around the pocket do not differ from the native CVB1 (Fig. 6D). This pocket has been previously targeted as a potential antiviral target for other CVBs and was shown to have a role in the stability of the capsid^45,46^.

Expanded CVB1-VLPs_Tween80_ can be recognized by the lack of density below the 5-fold seen in Fig. 5C,G,I and 6A. There is no density corresponding to VP4 of VP0 in the expanded CVB1-VLP_Tween80_ structure and no significant unmodeled density which would suggest a flexible region (Fig. 5C,G,I and 6A). Expansion at the 2-fold axis is consistent with expanded CVB1 A particle (PDB ID 7DQ4) as seen in Fig. 6F,G and the lack of lipid factor and a collapsed hydrophobic pocket are in line with the A-particle structure (Fig. 6C)^5^. A notable difference is in the presence of modellable VP3 loop comprised of residues 170–188 which is lacking in 7DQ4 and absence of the VP1 loop (197–204) which is present in 7DQ4 (Fig. 6F,G). Both models start at residue 58 of VP1 N-terminus (Fig. 6F,G). Residues on the VP3 loop are rotated away from the pore on the side of the 2-fold, potentially not blocking the VP1 N-terminus exit from the capsid (Fig. 6F)^5,8^. Unmodelled density shown at threshold of 1.5 *σ* above mean on the sides of the 2-fold outside the capsid could be flexible VP1 N-terminus (Figure S1B).

**Figure 7:**
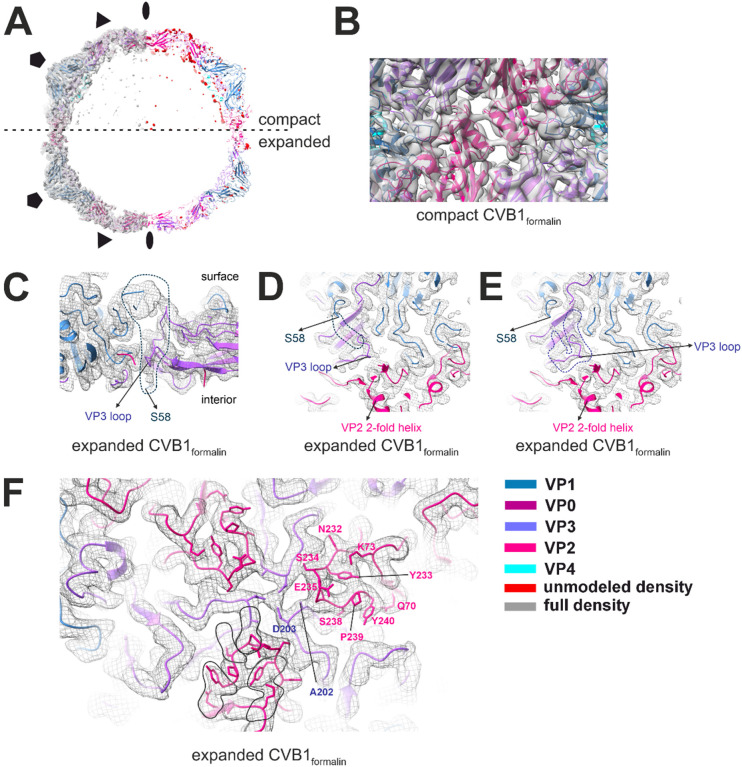
Final reconstruction analysis of compact and expanded CVB1_formalin_ obtained by cryoEM and single-particle reconstruction. A. Final reconstructions shown as surface at 2.5 (compact) or 5.5 (expanded) σ above mean with fitted model (left) and the atomic model with unmodeled density in red (right). Symmetry axes are marked with elipse (2-fold), triangle (3-fold) and pentagon (5-fold). Colour legend is in the bottom right corner of the figure. B. Focused view along the 2-fold axis of compact CVB1_formalin_ shown as model fitted into density map represented as surface at 2.5σ above mean. C-F. Focused view on the expanded CVB1_formalin_ shown as model fitted into density map represented as mesh at 5.5σ above mean. C. View through a slice of the capsid showing the beginning of VP1 N-terminus (S58) and VP3 loop at the quasi-3-fold pore. Density for possible VP1 N-terminus is marked with a dotted line and is clashing with VP3 loop. D and E. View of the quasi-3-fold pore with potential alternative densities which could be modelled marked with a dotted line for VP1 N-terminus (D) or VP3 loop (E). F. View on the surface of the capsid at the 3-fold symmetry axis. Density for ion is visible at the center of the 3-fold coordinated by 3 Asp. Cross-linking due to formalin treatment is circled in black on one asymmetric subunit. Residues of interest are marked on another asymmetric subunit. Cross-linking occurs both within and across different viral proteins.

Compact CVB1_formalin_ also exhibits structural characteristics of native CVB1, but due to the lower resolution at 4.1 A, the level of detail is insufficient for a thorough comparison (Figs. 5 and 7A,B, Table 1). Differences compared to compact CVB1VLP_Tween80_ are the lack of the density for a lipid factor inside the pocket and a strong, but non modellable density for VP1 N-terminus on the inside of the capsid below VP4 (Fig. 7A). Another distinctive difference is the presence of genome inside the capsid (Fig. 5O). Throughout the capsid, evidence of cross-linked residues could be observed, but were not modelled due to low resolution.

On the other hand, expanded CVB1_formalin_ is of sufficient resolution at 3.01 Å for a comparison at an atomic detail level to expanded CVB1-VLP_Tween80_ and CVB1 A-particles. Expanded CVB1_formalin_ shares the same characteristics as the other structures including expansion as noticed by the pore at the 2-fold, lack of a lipid factor and collapse of the pocket. Whether the VP1 N-terminus is exiting the capsid is unclear due to heavy crosslinking of the capsid and several potential densities it could occupy (Fig. 7C–E). VP3 loop, similar to a CVB1-VLP_Tween80_ structure, could occupy the quasi-3-fold pore and block the VP1 N-terminus, but the density for VP3 loop is weak compared to surrounding densities suggesting flexibility in the area. Furthermore, expanded CVB1_formalin_ lacks density for VP4 and RNA (Fig. 5P and 7A). As observed in the compact CVB1_formalin_ structure, heavy crosslinking is present throughout the capsid with residues contributing to most of the crosslinks being tryptophane, tyrosine and arginine and is occurring within and across different viral proteins (Fig. 7F). Similar to CVB1-VLP_Tween80_ structures, a density which could house an ion at the 3-fold symmetry axis is observed in expanded CVB1_formalin_ structure as well (Fig. 7F).

## Discussion

Adjuvants are generally categorized as immunostimulants and delivery systems and they are used to increase the immunogenicity of vaccines, especially in the case of protein-based vaccines. Certain adjuvants enhance immune activation by creating storage unit (depot) or immune niche for antigens at the injection site. This process may not only extend the period in which antigens remain within the immune system but may also attract additional immune cells to penetrate the injection site. This, in turn, may promote the absorption of antigens and trigger local inflammation, ultimately leading to the activation of subsequent adaptive immune responses. Previous studies have demonstrated that EGCG prevents CVB1-infection^21^, but the exact mechanism of action is not clear. One possible mechanism is binding of EGCG on the CVB1 capsid. In this study, we investigated the binding capacity of EGCG to CVB1-VLP and CVB1 particles with DLS analyses. Based on DLS-analysis, addition of EGCG to CVB1-VLP induced particle agglomeration, EGCG reversibly binding the capsids, forming over 1000 nm complex when EGCG was bound to the particle. Therefore, we hypothesized that the formed agglomerate may function as storage unit in the immunization site, and we experimented EGCG as a potential adjuvant for CVB1-VLP_No-Tween_ via the subcutaneous immunization route. Our study compared the adjuvant effects of commercially used AS04 to the effects of EGCG. EGCG exhibited promising adjuvant properties for CVB1-VLP_No-Tween_, as evidenced by higher levels of IgG1 and neutralizing antibodies (nAbs) compared to vaccines formulated with AS04 or without adjuvant (Fig. 2). This finding indicated the potential of EGCG as an effective adjuvant for enhancing vaccine-induced immune responses and warranted further investigation into its use.

In poliovirus, formalin inactivation has been shown to cause destruction of some immunogenic epitopes compared to live vaccines, although it still allows cell attachment, the virus is less likely to undergo the conformational changes associated with entry and release of RNA^44^. Although formalin-inactivated vaccines continue to be an important part of immunization schedules, the development of such vaccines requires growing the virus in large quantities in cell cultures, but this is difficult for several EVs due to their inefficient growth in standard cell culture systems. VLP vaccines can be generated for virus strains that do not readily grow in cell cultures. Perhaps most appealingly, VLPs are highly amenable to sequence modifications, enabling the introduction or omission of epitopes and the incorporation of stabilizing mutations, thus enhancing their efficacy and versatility^47,48^. VLP vaccines have already been successfully generated for other enteroviruses such as enterovirus 71, coxsackievirus A16 and A6 and enterovirus D68^48–58^.

Given the antiviral activity of EGCG against CVBs and other viruses^14–21^, we evaluated the applicability of EGCG for inactivating CVB1, producing traditional inactivated whole-virus vaccine. We hypothesized that the absorption of antigens may be enhanced through mucosa when EGCG attaches to CVB1. We demonstrated that EGCG bound to CVB1 virus, but infectivity decreased to the same level as formalin inactivated virus only after 1 month incubation at RT. Although EGCG required a longer incubation period compared to formalin (28 days compared to 5 days respectively), its antiviral activity could have been quicker if the virus would have been incubated with the compound at 37°C like for the formalin-inactivation and has been reported earlier^21^. To our knowledge, this study is the first to explore the use of CVB1-VLP or formalin inactivated CVB1 as a mucosal vaccine, although previous studies have tested the same strategy for formalin inactivated EV-71^59^, virus-vector based CVB3^60^, DNA based CVB3^61^ and an inactivated CV-A16 and CV-A16-VLP vaccines^62^. However, the previously described studies using inactivated virus vaccines in mucosal immunizations used adjuvants^59,62^.

Mucosal immunization represents a promising strategy for inducing robust immune responses at the site of viral entry. EGCG has been shown to be a successful adjuvant when administered in combination with ovalbumin as a model antigen^63^. Mucosal administration of EGCG together with the antigen showed an increase for mucosal IgA and systemic IgG, particularly IgG1^63^. Given our promising results on EGCG as a parenteral adjuvant, we investigated its efficacy in mucosal immunization using both CVB1-VLP_Tween80+EGCG_ and CVB1 inactivated with EGCG (CVB1_egcg_). Contrary to our expectations, EGCG formulation with CVB1 or CVB1-VLP_Tween80_ combined with intranasal immunization did not enhance the mucosal immunity against CVB1 or CVB1-VLP_Tween80_ in our preclinical model. Another study showed the co-administration of pro-vitamin A and EGCG with human immunodeficiency virus gp120 to have a synergistic effect enhancing the overall immune response. Interestingly and in line with our results, the intranasal administration of antigen administered only with EGCG without the pro-vitamin A, seemed to have a suppressive antigen-specific effect^64^. Although we were not able to enhance vaccine-specific immune responses with EGCG, the formalin-inactivated virus induced high antigen-specific humoral, cellular as well as mucosal immune responses. Most notably, high antigen-specific IgG- and IgA-antibodies as well as neutralizing antibodies detected in the BAL of vaccinated mice indicates that formalin-inactivated CVB1 functions as a promising mucosal vaccine inducing humoral, mucosal and cellular responses. Our hypothesis was that EGCG might stabilize certain CVB1 conformational epitopes and enhance mucosal vaccine uptake. However, our current findings challenge this hypothesis and indicate that the mucosal vaccine uptake could not be enhanced by EGCG. Based on our findings, the immunogenic antibody-specific epitopes of CVB1 may have been somewhat damaged when EGCG bound to the virus, because the humoral responses induced by the EGCG-inactivated virus are lower than those induced by formalin-inactivated virus. Interestingly, mice vaccinated with CVB1-VLP alone or formulated with EGCG intranasally induced weak antigen-specific IgG-, IgG1- and IgG2a-antibody responses in sera of vaccinated mice. This demonstrates that CVB1-VLP can be taken up by immune cells when administrated via mucosal route. However, new mucosal adjuvants need to be developed or identified for being able to induce more robust responses against VLPs.

We solved the atomic-resolution structure of CVB1-VLP_Tween80_ and CVB1_formalin_ that were used in the immunizations. Such knowledge was needed to be able to compare the particle structure and immunogenicity and to facilitate future modifications and optimizations producing more efficient VLP vaccines. Through cryoEM and single-particle image processing, two distinct particle populations were separated. These particle populations were termed compact and expanded, structurally corresponding to mature native CVB1 virions and CVB1 A-particles respectively^5^. Previously, it has been shown that both of these particle conformations can bind CVB1-specific neutralizing antibodies, demonstrating that at least the conformations of the analysed neutralizing-antibody epitopes were not affected by the viral allosteric process^65^. Here we observed two structural differences of CVB1-VLPs_Tween80_ compared to native CVB1 which are the presence of a VP3 loop in the pore of the expanded CVB1-VLPs_Tween80_ and an additional density in the interprotomer pocket of compact CVB1-VLP_Tween80_. Both are unlikely to interfere with antibody binding and eliciting a lower immune response.

We demonstrated that the distribution of the particle populations is 85–15% in favour of the expanded particles in formalin-inactivated virus, whereas the ratio was 48% compact to 52% expanded for VLPs, demonstrating a marked difference in the proportions between the vaccines. In contrast to earlier studies with poliovirus where formalin inactivation prevent expansion^44^, in our preparation, expansion and loss of genomic RNA occurred, yet immunogenicity was still good. This agrees with work by Zheng et al on CVB1 nAb^65^ which showed the ability of three neutralizing antibodies to bind to all conformations of CVB1, including empty particles and A-particles, and compete with the receptor for binding while inducing high immune response^65^. The same observation was made with Enterovirus A71 VLPs^66^. Interestingly, nAb 8A10 studied in^65^ has an epitope at the same site where the additional density was observed in the interprotomer pocket of compact CVB1-VLP_Tween80_. Therefore, it is unlikely that the additional density would block the antibody binding due its small size, but it is worth a mention and can allude to the importance of VLP expression system and purification conditions. However, here the potential reason for the lower CVB1-VLP_Tween80_ immunogenicity compared to formalin inactivated virus is the lack of RNA in the VLP. Differences of CVB1_formalin_ and native CVB1 include heavily cross-linked capsid and potential internalisation of VP1 N-terminus in expanded CVB1_formalin_. Internalized N-terminus would not be accessible to antibodies and may elicit a different response than that of expanded CVB1-VLPs_Tween80_. Furthermore, none of the cross-linked sites coincide with antibody epitopes discussed in Zhen et al.^70^ Which form of the particles is responsible for the immune response is not known, but the presence of a large population of expanded particles does not seem to hinder the vaccine potential of formalin inactivated virus. Despite the lower immunizing properties, VLPs have shown to be stable for at least one month and can handle freezing and thawing while retaining almost 50% of the particles in the compact form.

In conclusion, our results highlight the potential of using formalin inactivated CVB1 vaccine in mucosal immunization programs. However, as traditional manufacturing cannot be applied to several enteroviruses (or other viruses), our results highlight the importance of further development for next-generation VLP-based vaccines. The atomic resolution structural data presented here for CVB1-VLP enables further developments for the candidate vaccine holding a promise for developing vaccine platform that can be applied for all enteroviruses.

## Figures and Tables

**Table 1:** Cryo-EM data collection, refinement and validation statistics

		compact CVB1-VLP_Tween80_	Expanded CVB1-VLP_Tween80_	compact CVB1 _formalin_	expanded CVB1 _formalin_
Data collection and processing					
Magnification		105000	105000	150000	150000
Voltage (kV)		300	300	200	200
Electron exposure (e-/Å^**2**^)		63.368	63.368	40	40
Defocus range (μm)		−0.2 to −1.6	−0.2 to −1.6	−0.1 to - 2.8	−0.1 to - 2.8
Pixel size (Å)		1.06	1.06	0.97	0.97
Symmetry imposed		I2	I2	I2	I2
Micrographs (no.)		13860	13860	615	615
Initial particle images (no.)		108270	117953	289	1935
Good particles		86988	117738	289	1935
Final particle images (no.)		86988	117738	289	1935
Map resolution (Å)		2.15	2.15	4.10	3.01
FSC threshold		0.143	0.143	0.143	0.143
Map resolution range (Å)		999 – 1.94	999 – 1.94	999 – 1.94	999 – 1.94
Refinement					
Map sharpening *B* factor (Å^**2**^)		−56.2	−65.1	−32.6	−100.7
Model composition					
Non-hydrogen atoms					
Protein residues					
VP1		56–278	58–197, 203–277	yes	58–197, 204–277
VP0	**VP2**	13–263	13–263	yes	12–43, 54–260
	**VP4**	27–43	no	yes	no
VP3		1–238	1–232	yes	1–232
Ligands		Lipid, ?			
R.m.s. deviations					
Bond lengths (Å)		0.29	0.33	-	0.33
Bond angles (°)		0.48	0.50	-	0.50
Validation					
MolProbity score		1.55	2.27	-	2.27
Clashscore		3.36	5.84	-	5.84
Poor rotamers (%)		2.97	6.48	-	6.48
Ramachandran plot					
Favored (%)		97.78	95.12	-	95.12
Allowed (%)		2.22	4.29	-	4.29
Disallowed (%)		0	0.59	-	0.59

## Data Availability

The datasets used and/or analyzed during this study are available from the corresponding author on reasonable request. The cryoEM densities are deposited in the wwPDB with accession numbers 9FJC for compact CVB1-VLP_Tween80_, 9FJD for expanded CVB1-VLP_Tween80_ and 9FJE for expanded CVB1_formalin_. Sequence for the CVB1–10796 can be accessed in GenBank with accession number PP782006.
